# Practical Tips for Starting a Student-Run Free Eye Clinic

**DOI:** 10.12688/mep.20621.1

**Published:** 2024-10-02

**Authors:** Robert Porter, Adam Leone, Cameron Reinisch, Alec Murphy, Courtney Linne, Jonathan Regenold, Hyun Jun Kim, Lisa Kelly

**Affiliations:** 1 University of Cincinnati College of Medicine, Cincinnati, Ohio, 45267, USA

**Keywords:** medical student education, outreach, student-run free clinic

## Abstract

Student-run free clinics serve three primary functions: 1) providing basic medical services for the uninsured and underserved in our communities, 2) supporting the training of medical providers, and 3) advocating for medically underserved communities. Despite the multiple benefits that student-run free clinics can provide, the process of founding a clinic is a significant challenge, even for highly motivated students.

Here, we present 12 tips generated by students and residents at the University of Cincinnati College of Medicine based on their combined experience in founding the Bearcat Eye Service (BES), a clinic that provides basic ophthalmic screenings. These tips were developed to provide a framework for those interested in founding a student-run free clinic or expanding ophthalmic care in their communities.

## Introduction

Student-run free clinics (SRFCs) provide valuable services to the local community, such as social services and screening and education for common medical conditions. SRFCs also provide opportunities for medical students to exercise their medical knowledge and provide patient-centered and culturally sensitive care. Indeed, many students consider founding a student-run free clinic during medical school, and studies show they fill a critical niche by delivering care to patients who would often go without care otherwise ( Rupert *et al.,* 2022). When it comes to eye conditions, only 23 of the 106 SRFCs in the United States provide screening and education for ophthalmic diseases, such as cataracts, glaucoma, and diabetic retinopathy, despite the disproportionate health and economic burdens borne by those of socioeconomic disadvantage ( Okaka *et al.,* 2021; Wittenborn *et al.,* 2013).

Herein, we present practical tips generated by medical students and residents at the University of Cincinnati College of Medicine (UCCOM) that founded Bearcat Eye Service (BES), a clinic that provides basic ophthalmic screenings in the local community. BES has three primary functions: 1) to provide ophthalmic screening and education to those of socioeconomic disadvantage, 2) to provide medical students a hands-on way to learn about the field of ophthalmology, and 3) to contribute to the national conversation of community service and health through research. While these tips are arranged chronologically, many of the steps can occur simultaneously. While some tips are specific to ophthalmology, most can be utilized by those who desire to start an SRFC in any capacity.

### Tip 1: Collaborate with local and national organizations

Develop partnerships with trusted organizations to unlock key opportunities and relationships. Early on, founding members of BES traveled to the University of Pittsburgh to learn about their student-run free eye clinic, the Guerilla Eye Service ( Bonilla-Escobar, 2017). The founding members also traveled to the University of Miami Department of Community Service Programs to learn about how medical students collaborated with residents to provide care for the community ( The Mitchell Wolfson Sr. Department of Community Service, 2024). Subsequently, the BES partnered with another medical student organization, the University of Cincinnati Student-Run Free Clinic (UC-SRFC), which provided several advantages. First, it allowed the BES to serve an already-defined patient population with clear medical needs. Second, it allowed the BES to take advantage of the established relationship of trust that the UC-SRFC had with UCCOM medical students and attendings. Last, the BES could then join existing medical-legal agreements established by the UC-SRFC and utilize their medical record system.

There are also national consortiums that can be beneficial. The Society of Student-Run Free Clinics conference and the Community Health Leadership Conference (hosted by the Department of Community Service at the University of Miami Miller School of Medicine) are annual conferences that provide opportunities for collaboration and discussion among student leaders from institutions across the country. Both conferences allow for oral and poster presentations but also include addresses from keynote speakers and collaborative sessions where students can share ideas and discuss ways they can improve the delivery of community service at their institutions. Additionally, the BES partnered with Prevent Blindness, a nonprofit that offers a free Adult Vision Screening Certification program. The program provides vision screening training and a free Snellen chart to those planning to provide their screenings in Wisconsin or Ohio ( Prevent Blindness, 2024). The training will also help your organization get ahead of a continual challenge you will face as medical student classes progress year-to-year: training new groups of volunteers to operate the clinic independently.

### Tip 2: Identify a location for the clinic

Select an accessible location for your clinic. The UC-SRFC operates within the Healing Center, a local non-profit that provides food services, educational classes, healthcare, and other community resources. A key reason for partnering with the UC-SRFC and the Healing Center was a clean location near a major highway with access to public transportation, and the space for an eye clinic to operate. Another advantage of the location is that patients could take advantage of the other services offered at the Healing Center while being seen at the BES.

Accessibility is a fundamental concept in health policy, and the leaders of your clinic should strive to choose a location close in proximity to public transport routes or major highways ( Penchansky & Thomas, 1981). It can be helpful to reach out to pre-existing organizations, whether associated with your institution or the community, to see if they are familiar with a suitable location. Schools and places of worship are often great places to start since they tend to be clean, safe, and motivated by a similar desire to help others. It is also important to understand how much space your free clinic will need and if your desired location can accommodate that ( American Medical Association, 2016). Acquiring a location with 2–3 private rooms for patient interactions is essential for creating a space where patients can feel comfortable and safe. Make sure to consult with legal experts at your institution to understand any liability issues that may be associated with choosing your desired location.

### Tip 3: Conduct a needs assessment for the target community

Perform a needs assessment to help identify barriers to care specific to the community. The BES designed and implemented a Community Health Needs Assessment (CHNA), a widely accepted tool used by both clinics and hospitals to serve the broad interests of the community ( Ravaghi *et al.,* 2023). The CHNA was implemented at the Healing Center. For the BES, the findings of a needs assessment were a key component in securing funding. It also demonstrated dedication to a mission, which solidified our partnership with the Healing Center.

Consulting with professional and federal recommendations when designing the needs assessment is essential. When designing our CHNA in 2021, we consulted a published CHNA ( Haller *et al.,* 2016), as well as other tools published by the CDC ( Centers for Disease Control and Prevention, 2019), WHO ( World Health Organization, 2022), and the NHS ( Cartwright *et al.,* 2018). Conducting a CHNA protocol will require approval by your Institutional Review Board (IRB) and can generally be formatted to meet the requirements for the non-human subjects research designation ( U.S. Department of Health and Human Services, 2018). The results of our CHNA were later presented at the 2022 National Society of Student Run Free Clinics Conference in Mobile, Alabama ( Linne *et al.,* 2022). Needs assessments are important to identify barriers to care and to introduce you to other community organizations and representatives of the community, who can help elucidate the community needs. Partnering with these existing community organizations and representatives will aid in garnering the trust of the community and in implementing your services ( U.S. Internal Revenue Service, 2024).

### Tip 4: Seek out a champion

Identify a faculty attending to be the champion for the SRFC. A champion is usually a high-ranking attending physician with values that align with your organization’s mission, and they will be able to advocate for your organization at the institutional level. The BES faculty champion assisted in acquiring grant funding, navigating medical-legal questions, and submitting our work to conferences and journals.

Medical students need to appreciate the significant responsibility and time commitment taken on by the champion. In exchange for the champion’s support, medical students must work in a manner that respects the name and reputation of that champion. In communicating with the attending, keep channels of communication open but professional. Do your best to make the lives of your supporters as easy as possible and make sure that they are regularly updated on the status of current projects, so they are not surprised. To achieve this, the BES has regular executive meetings and sends monthly project updates to all attendees involved with BES with the status and planned next steps, broken down by project category (e.g., research, clinic operations, and fundraising).

### Tip 5: Utilize the needs assessment to generate a focused scope of practice

Generate a focused scope of practice using data from your needs assessment that addresses the needs and barriers faced by your community. Before conducting the needs assessment, the BES considered providing several services including basic eye care, surgical referrals, and eyeglasses prescriptions. However, when our needs assessment identified that many Spanish-speaking individuals did not have insurance and had never had an eye exam, we decided to initially focus on the screening of common ophthalmic conditions. This focused scope determined how we market ourselves to potential community partners, facilitated efficient communication with attending physicians, and helped make the most out of the limited time and resources of medical students and residents.

Inevitably, there will be valuable services that fall outside of your focused scope of practice. The decision to forego these benefits may feel like betraying the spirit of community service, which draws many to take on the endeavor of starting an SRFC. However, a focused scope of practice will be instrumental in getting the clinic open in a timely fashion. It will also keep work at a manageable level for medical students whose primary goal is to learn the foundations of medicine. The scope of practice can always be expanded in the future.

### Tip 6: Acquire mentors who help make decisions, advocate for you, and are willing to volunteer

Find mentors who can hone the vision of medical students and provide guidance on the practical implementation of clinical principles. The BES was founded by ophthalmology residents supportive of medical students interested in ophthalmology and community engagement, which made them natural mentors for the organizations. However, attendings and residents can be recruited to provide additional support.

Good communication skills are essential to communicate students’ dedication clearly and concisely to potential mentors. For best success and to demonstrate dedication to their goal, it is often beneficial for medical students to develop a formal proposal and clinic plan for their potential mentor. In writing proposal emails, aim to be polite and concise, and have actionable steps for which mentors can reasonably assist. In addition, supply your mentors with clear deadlines that are easily met. These principles can also be applied when reaching out to attending physician volunteers. Ideally, these volunteers should have prior experience working with student-run free clinics and be willing to staff clinic days. They will be able to advise your organization on the necessary equipment and know which screening procedures are most critical. Additionally, if available at your institution, it is helpful to have resident physicians staff the clinic for more advanced procedures and to aid in medical student learning.

### Tip 7: Recruit a diverse group of dedicated student volunteers

Enlist volunteers from your institution’s medical student population who are dedicated to caring and advocating for medically underserved communities. A group of founding medical students were highly involved in the creation of BES, with the assistance of faculty and resident mentorship. These students continue to manage behind-the-scenes operations of BES including fundraising, research, clinic flow, and patient navigation. Once the BES began seeing patients, additional medical student volunteers were recruited with a focus on finding a diverse group of students who are dedicated to advocacy and patient-centered care.

It is crucial to recruit enough medical students, as they will provide the necessary manpower to operate your organization. Determining how many volunteers are sufficient may change over time based on the scope of your clinic. At the start, it will be based on the results of your needs assessment, the number of clinic days per month, number of patients seen, etc. Your organization may also benefit from the recruitment of undergraduate students and other healthcare professionals, such as physician assistants, nurses, and pharmacists ( Darnell, 2010). Enabling a multidisciplinary approach will enhance the quality of care provided to your patient population.

### Tip 8: Implement fundraising and grant-writing initiatives

Create a dedicated fundraising and grant-writing committee. The BES created this committee and tasked the members with creating a comprehensive budget, strategically outlining essential spending, and identifying and applying for appropriate grants. The award of multiple key grants allowed for the BES to obtain costly ophthalmic equipment, and continues to fund clinic operations, research projects, and patient outreach.

SRFCs have limited financial resources, and some label funding as one of their biggest challenges ( Smith *et al.,* 2014). Applying for grants is an important way to address this challenge. One way to search for grants is to reach out to similar local, state, or national organizations to see what grants they are applying for. Clinics can also utilize databases that help connect them to eligible grants. Candid, for example, is an organization that provides comprehensive data for non-profits ( Candid, 2024). Some groups that give grants do not award grants to new organizations, as they like to see these organizations acquire money first through fundraising. Clinics such as the Morehouse School of Medicine Health Equity for All Lives (MSM-HEAL) student-run clinic have documented success through annual fundraising events, networking with stakeholders and community leaders, and alumni and faculty donations after sharing their stories with them ( Stubbs *et al.,* 2019).

### Tip 9: Choose a type of medical record

Determine which medical record is best for your clinic. Multiple types of medical records were considered for use by the BES, including commercially available electronic medical records (EMRs), online data storage solutions, and paper charts. The faculty champion was instrumental in coordinating with the legal team to determine which type was most appropriate. The BES decided to utilize a paper charting system with HIPAA-compliant storage, and the paper chart format was created under the guidance of faculty mentorship. The secure storage system was already developed by the UC-SRFC.

Cost is probably the biggest factor in whether an SRFC can use a specific EMR. Commercially available EMRs like EPIC are user-friendly and connect naturally with the hospital but are often prohibitively expensive. One such EMR, athenaOne, is free for 501©(3) organizations through the athenaGives program ( Athenahealth, 2024). Other affordable options include 75 Health and OneTouch EMR (free with the ability to purchase specific features). Clinics that are a part of the National Association of Free Clinics are also provided free access to Practice Fusion as a part of their annual membership fee ( Practice Fusion, 2024). Ease of use is also an important feature for an SRFC that has a rapid turnover of students. Google Sheets provides this feature, and it can be made HIPAA-compliant by entering into a HIPAA Business Associate Addendum (BAA) with Google. We recommend consulting your legal and IT experts in following the manual that Google provides to accomplish this ( Google, 2024). RedCap is typically used for surveys and research, but it is also HIPAA-compliant, and some clinics utilize it for this purpose as well. Paper documentation is cheap and can be used without internet access or paying for an EMR, but it does require protocols for restricted access, storage, and disposal. Best practice involves plans to restrict chart access only to relevant providers on clinic days, the “HIPAA double lock policy” of keeping files in a locked file drawer or safe in a locked room, and routine condensing and destruction (e.g. via shredding) of redundant or unnecessary records ( Yale University, 2024).

### Tip 10: Acquire the appropriate equipment for clinic operations

Obtain medical equipment that aligns with the focused scope of your clinic. Discussions with faculty mentors as well as students working with similar clinics helped generate a list of equipment that would serve the scope of our clinic. Grant funding obtained by the fundraising committee was utilized to purchase the equipment via a local ophthalmic instrument sales contractor.

To acquire advanced machinery for your clinic, start by reaching out to the independent contractors that provide such machinery to your affiliated hospital. They should have access to channels that could provide used equipment at discounted rates or as donations (as for the latter, be sure to be partnered with an organization that can legally receive donations so that you may reciprocate the gift of donated equipment with the potential for a tax benefit for the donor). Not only might these independent contractors be able to help with equipment acquisition, but they would also be equipped to test the machines’ functionality, calibrate as necessary, and provide insight into the optics and dynamics of testing room setup. Another avenue to pursue is connecting with organizations that provide surplus medical equipment, such as the Med Surplus Alliance. To qualify for equipment, an application is required, and your organization must be qualified to accept duty-free humanitarian aid.

### Tip 11: Establish a process for patient referral and follow-up

Build a protocol for patient referral and follow-up before the clinic opens. To facilitate referrals, the BES has a patient navigation committee which is responsible for proactively reaching out to patients, offering reminders of upcoming appointments, and addressing any potential barriers that may hinder attendance. A referral and the respective patient information are transferred via secure fax to the UC Health Ophthalmology resident clinic when patients need medical and/or surgical treatment. In addition, patients are provided with a UC Health financial assistance application which they can submit at the UC Health financial aid office. Members of the patient navigation committee then reach out by phone to ensure patients have an appointment scheduled and have reliable transportation.

Past experiences from other SRFCs have shown that only 19.4% (35 out of 180) of patients successfully attended their follow-up appointments, with an average interval between visits of 14.4 months ( Scheive *et al.,* 2022). Despite the efforts by the BES to address this disparity, some challenges have arisen in facilitating patient follow-up. One challenge is communication with the patient after their visit, due to difficulties with technology literacy and/or lack of reliable access to lines of communication including phone or email. Another challenge is the lack of transportation to appointments, or to the UC Health financial aid office since patients often do not have the required paperwork on hand during their visit to complete the financial aid application. Flexibility with your clinic structure and utilization of your mentors’ guidance will assist you in tackling obstacles that arise.

### Tip 12: Build for patient-centered care

Ensure that patient-centered care is a core principle of your clinic. Feedback from patients through the CHNA conducted by the BES highlighted language barriers and medical literacy as significant barriers to care specific to the community. To address these barriers, the BES partnered with the UCCOM Medical Spanish/Latino Health Elective to deliver culturally proficient and linguistically competent care to the Cincinnati Latino community. Printed educational materials were translated into Spanish, and medical students with interpretation abilities were recruited as clinic volunteers. After officially opening, the BES developed processes for patients to provide feedback. Monthly meetings with BES volunteers and executive leadership actively utilize this feedback to shape the services we build. For example, after identifying transportation as a barrier to establishing care at the UC Ophthalmology clinic, grant funds were diverted to provide gift certificates for ridesharing services to help patients make their medical follow-up appointments.

The goal of patient-centered care is to empower patients to become active participants in their healthcare, hoping to better secure a good outcome ( Reynolds, 2009). We believe that having patient-centered care as a core principle of your clinic from its inception will allow you to best serve your target community, highlighting the importance of incorporating patient feedback in real-time.

## Conclusion

Opening a student-run free clinic is an opportunity to give back to your community and provide crucial screening for patients who may not otherwise get the care that they need, while also empowering patients to actively participate in their care. Obtaining the necessary equipment, funding, and support to open these clinics may seem like a daunting task, but we hope that these practical tips can simplify this process by providing a framework for you to do so. Having a focused, goal-directed approach in conjunction with institutional and mentor support is invaluable in getting the first steps completed. Obtaining the necessary funding, equipment, and volunteer manpower are goals that are necessary but will be much more readily achieved with the appropriate guidance and expertise of your mentors and champion. BES has had initial success in seeing patients, and we are continually expanding our scope as appropriate while ensuring patient-centered care is one of our core priorities. We hope that these tips have sparked interest in existing SRFCs to expand their services to include eye care.

## Ethics and consent

Ethical approval and consent were not required.

## Data Availability

No data are associated with this article.
