# Supplementary material for: Practical Tips for Starting a Student-Run Free Eye Clinic
Source: MedEdPublish (2016). 2025 Feb 27;14:207. Originally published 2024 Oct 2. [Version 2] doi: 10.12688/mep.20621.2 (PMC12231372; doi:10.12688/mep.20621.2)
Supplement: Peer review history

# Reviewer report: Reviewer response for version 2

**DOI:** 10.21956/mep.22443.r41140

**Published:** 2025-03-06

**Authors:** Marek Karas

**Affiliations:** 1 Cardiff University, Cardiff, Wales, UK

Thank you for asking me to review this revised version. I am happy that the authors have addressed my concerns and I have no further comments.

If evidence from practice is presented, are all the underlying source data available to ensure full reproducibility?

Not applicable

Is the topic of the practical tips discussed accurately in the context of the current literature

Partly

Are all factual statements correct and adequately supported by citations?

Yes

Are the conclusions drawn balanced and justified on the basis of the presented arguments?

Yes

Are arguments sufficiently supported by evidence from the published literature and/or the authors’ practice?

Partly

Reviewer Expertise:

Optometry education, pediatric eye care and eye in learning disabled populations.

I confirm that I have read this submission and believe that I have an appropriate level of expertise to confirm that it is of an acceptable scientific standard.

# Reviewer report: Reviewer response for version 1

**DOI:** 10.21956/mep.22074.r39411

**Published:** 2024-11-25

**Authors:** Marianne E F Piano

**Affiliations:** 1 University of Surrey, Guildford, England, UK

Thank you for the opportunity to review this paper which provides practical advice for setting up a student-run free eye clinic. There is a lot of helpful information and it is appropriately referenced.

 While there are a wealth of practical tips buried within this article, much of the content is not that specific to the ophthalmology setting, dealing with matters general to all healthcare clinics such as patient data protection, procurement of equipment, identification of a faculty champion, access to tertiary services after referral, and other such details.The manuscript could be enhanced by more specific considerations such as that detailed in Tip 5 regarding the need for screening. This could be literature-based, such as with reference to US epidemiological data about common eye conditions experienced by Latinx and Hispanic populations. Similarly Tip 12 can be more based in the literature regarding health inequalities experienced by this population and how reduced health literacy can impact eye health outcomes. Tip 9 could also consider some of the more specialised aspects of ophthalmic screening recording, such as what visual acuity test was used.

 Lastly, it would be helpful to know if the students received any support to be part of this clinic e.g. a clinic orientation, education about working with culturally and linguistically diverse populations and other such considerations.

If evidence from practice is presented, are all the underlying source data available to ensure full reproducibility?

Not applicable

Is the topic of the practical tips discussed accurately in the context of the current literature

Yes

Are all factual statements correct and adequately supported by citations?

Yes

Are the conclusions drawn balanced and justified on the basis of the presented arguments?

Yes

Are arguments sufficiently supported by evidence from the published literature and/or the authors’ practice?

Yes

Reviewer Expertise:

clinical eyecare

I confirm that I have read this submission and believe that I have an appropriate level of expertise to confirm that it is of an acceptable scientific standard, however I have significant reservations, as outlined above.

# Reviewer report: Reviewer response for version 1

**DOI:** 10.21956/mep.22074.r39532

**Published:** 2024-11-14

**Authors:** Marek Karas

**Affiliations:** 1 Cardiff University, Cardiff, Wales, UK

**Practical Tips for Starting a Student-Run Free Eye Clinic**

 Thank you for asking me to review this paper. I feel this paper is a useful addition to the literature on volunteer student teaching clinics. I have no major concerns to voice but do have some minor suggestions that I feel will improve the paper for the reader.

 I feel that a clearer understanding of the organisational structure of the clinic would be useful covering both institutional and clinical oversight. Some of this information is covered within the twelve sections but it would be useful to see it in summary. This might be included as a figure.

 Last sentence first paragraph.  This needs to be looked at. The Wittenborn paper looks at the socioeconomic burden borne to society for those under 40’s but it does not look at the burden by economic group. Either the statement needs to be revised or an alternative reference found.

 Tip 2. Second paragraph Line 7. I understand that this paper is to some extent informal and this is of course an editorial decision but the term “great places to start” is too informal in my view.

 Tip 3 First paragraph Line. The CHNA method as detailed by the Ravaghi review determines the needs of a community rather than “serves the interest of”.

 Tip 6. First sentence. “hone the vision of medical students” seems a bit esoteric and wide ranging. It would be better to be more specific about the role of the mentor and this links to my initial comment about setting out the roles of the various stakeholders.

 Tip 6 Second paragraph. “often beneficial” is too vague in this context. I think the authors are saying that they found is beneficial to clearly set out a proposal covering organisational and clinical areas.

 Tip 7. Second paragraph Line 6. “etc.” is too vague in a paper.

 Tip 8. Second paragraph Line 9. The statement about groups giving grants needs to be put into the context of a reference or the experience of the authors. “In the authors experience….”

 Tip 9. HIPAA-compliant storage needs to be defined and referenced.

 It would be useful to understand how the clinic fitted into onward referral. I understand that the paper is not describing the clinic itself and its outcomes but in setting up such a service how it links into onward services is a key consideration.

If evidence from practice is presented, are all the underlying source data available to ensure full reproducibility?

Not applicable

Is the topic of the practical tips discussed accurately in the context of the current literature

Partly

Are all factual statements correct and adequately supported by citations?

Yes

Are the conclusions drawn balanced and justified on the basis of the presented arguments?

Yes

Are arguments sufficiently supported by evidence from the published literature and/or the authors’ practice?

Partly

Reviewer Expertise:

Optometry education, pediatric eye care and eye in learning disabled populations.

I confirm that I have read this submission and believe that I have an appropriate level of expertise to confirm that it is of an acceptable scientific standard, however I have significant reservations, as outlined above.

# Reviewer report: Reviewer response for version 1

**DOI:** 10.21956/mep.22074.r39410

**Published:** 2024-11-06

**Authors:** Jaymie T Rogers

**Affiliations:** 1 The University of Auckland, Auckland, New Zealand

Thank you for the opportunity to review this article. This topic is timely and significant, given the increasing prevalence of vision impairment worldwide. In summary, this manuscript presents twelve recommendations to successfully establish a student-run free eye clinic. For each recommendation, the authors offer a practical rationale for their suggestion and include an example from their own clinic.

 Some suggestions to enhance the clarity of the information:

- In the introduction, please consider briefly outlining the structure of the BES clinic to provide context for the tips and rationale presented later. For example, "Institutional support" is mentioned in the conclusion, providing an early explanation of the supervisory role within the clinic would help readers understand how these student-run clinics operate or are over-seen.
- Additionally, since Tip 8 addresses funding initiatives, a brief mention in the introduction, of how the BES clinic is financed (whether through institutional backing, grants, or donations) would offer valuable insight into the clinic's setup and sustainability.
- Tip 4 to 6 offer valuable recommendations, please consider incorporating supporting evidence from published literature to strengthen these recommendations and statements. 

If evidence from practice is presented, are all the underlying source data available to ensure full reproducibility?

Not applicable

Is the topic of the practical tips discussed accurately in the context of the current literature

Yes

Are all factual statements correct and adequately supported by citations?

Partly

Are the conclusions drawn balanced and justified on the basis of the presented arguments?

Yes

Are arguments sufficiently supported by evidence from the published literature and/or the authors’ practice?

Yes

Reviewer Expertise:

Community eye health research,  access to eye care for underserved communities.

I confirm that I have read this submission and believe that I have an appropriate level of expertise to confirm that it is of an acceptable scientific standard.
